# Temporal Trends in Pancreatic Cancer in New South Wales, Australia: A Longitudinal Population‐Based Study of Incidence, Surgery and Survival

**DOI:** 10.1111/ans.70559

**Published:** 2026-02-26

**Authors:** Joshua Mok, Jacqueline Close, Robert Gandy, Lara Harvey

**Affiliations:** ^1^ School of Clinical Medicine, UNSW, Prince of Wales Clinical School Sydney Australia; ^2^ NeuRA, Falls, Balance and Injury Research Centre Sydney Australia; ^3^ School of Population Health, University of New South Wales Sydney Australia

**Keywords:** incidence, New South Wales, pancreatectomy, pancreatic carcinoma

## Abstract

**Introduction:**

The incidence of pancreatic adenocarcinoma is increasing, as is the proportion of patients undergoing pancreatectomy. This study aimed to identify temporal trends in incidence, survival and pancreatectomy uptake in New South Wales (NSW), in patients aged 50 years and older diagnosed with pancreatic and periampullary cancer.

**Methods:**

This study linked NSW Hospitalisation, Death, and Cancer Registry data. Analysis included patients aged 50 years and older diagnosed or admitted for pancreatic or periampullary cancer (2009–2018). We analysed temporal trends in incidence and age‐standardised rates of pancreatic cancer, pancreatectomy rates, and 1‐, 3‐ and 5‐year post‐diagnosis survival.

**Results:**

Thirteen thousand five hundred and sixty patients were identified, with annual cases increasing from 1148 to 1591. Age‐standardised pancreatic cancer rates increased from 50.3/100 000 to 56.3/100 000 population, an annual increase from 2009 to 2018 of 1.5% (95% CI 1.0–2.0). From 2009 to 2018, one‐ (27.7% vs. 39.7%) and three‐ (13.8% vs. 20.9%) year survival improved, and an increased proportion of patients underwent resection (14.5% to 21.4%). Five‐year survival similarly improved (11.6% vs. 19.0%) between 2009 and 2016. Pancreaticoduodenectomy rates decreased, and non‐pancreaticoduodenectomy resection rates increased. An increased proportion of pancreatectomies were conducted in high volume centres (≥ 16 pancreatectomies). Diagnosis in a later year improved survival in both the whole, and the surgical cohorts at all endpoints.

**Conclusions:**

In NSW, rates of pancreatic and periampullary cancer in people aged 50 years and older have risen. Despite remaining a disease of high mortality, survival has improved over a 10‐year period, and resection is increasingly utilised.

## 1 Introduction

Pancreatic cancer is an aggressive and frequently fatal malignancy with a growing incidence internationally [1]. Between 1990 and 2017, the worldwide age‐standardised incidence increased from 5.0 (4.9–5.1) to 5.7 (5.6–5.8) per 100 000 population [2]. Pancreatic cancer is the eighth most common cancer in Australia and the third most common cancer leading to death [3]. A disease of older populations, around 70% of individuals are diagnosed over the age of 65 years [4]. An ageing population and changing prevalence of risk factors such as diabetes and obesity increase the incidence of pancreatic and periampullary cancer [1, 2, 3].

Consistent with other high‐income countries, 5‐year survival for pancreatic cancer in Australia is around 10% (10.7%; 2012–2016) [3, 5, 6]. Five‐year survival for pancreatic cancer patients diagnosed at 80 years and older decreases to 2.9% [3]. Previous studies have observed improved survival associated with changing practices around systemic therapy, surgical selection, perioperative care and regionalised models [5, 7].

Pancreatectomy in combination with modern systemic therapy offers the only chance for long‐term survival in pancreatic cancer. In Australia, only 15% of patients with pancreatic cancer undergo resection, largely due to advanced stage of disease at presentation, and being inappropriate for major surgery due to comorbidities and frailty [8, 9, 10]. Whilst the proportion of older patients undergoing pancreatectomy is increasing, few people older than 80 years (7.5%) [10] undergo pancreatectomy. This is likely attributable to comorbidity and frailty, both of which are associated with ageing, and an increased risk of poor surgical outcomes [9, 11]. In Australia, the number of pancreatectomies for all indications in people aged 50 years and older was 1558 in 2020–21 [12].

Increasing treatment centralisation and changing guidelines around the management of pancreatic and periampullary disease may improve access to pancreatic resection, likely influencing patient outcomes [13, 14]. The Cancer Institute NSW supports minimum hospital volume standards at six pancreatectomies per year [15]. Increasing use of neoadjuvant therapy, which may offer individuals previously not considered for pancreatectomy a chance at operative intervention, may contribute to increasing resection rates [13, 14, 16]. Guidelines on pre‐operative optimisation of comorbidities [17], and growing consensus on the use of extended resections [18] may also contribute to increased resection rates.

In an Australian context, little is known about the changing incidence, resection rates and outcomes of pancreatectomy for pancreatic and periampullary cancer. The Australian Institute of Health and Welfare (AIHW) reports trends in rates of pancreatic cancer; however, it does not report on resection and patient characteristics. A single previous study reported on pancreatic cancer and pancreatectomy in New South Wales (NSW) between 2005–2013 and found that pancreatectomy was likely underutilised in several regions within NSW, highlighting systemic inefficiencies and access issues [19]. This population‐based, retrospective cohort study explores temporal trends in incidence, intervention, and survival amongst pancreatic and periampullary cancer patients in New South Wales, Australia.

## 2 Methods

### 2.1 Cohort

The study cohort included all adults aged 50 years and older with a diagnosis of, or hospital admission for, pancreatic or periampullary cancer in NSW, Australia over the 10‐year period 1 January 2009 to 31 December 2018. NSW is Australia's most populous state, with over 8 million individuals, of whom 2.8 million are individuals aged 50 years and older (35.8%) [20]. The median age in NSW is 39 years. Data was drawn from the NSW Central Cancer Registry (CCR) and the Surgical Care of Older People (SCOPE) dataset. The SCOPE dataset comprises linked de‐identified hospital (Admitted Patient Data Collection (APDC)), death (Register of Births, Deaths and Marriages (RBDM) and Cause of Death—Unit Record File (COD‐URF)) data for all patients aged 50 years and older, admitted to NSW public and private hospitals under a surgical team from 1 January 2007 to 31 December 2021.

The APDC records NSW hospitalisations and provides information on demographics, diagnoses, and procedures, coded according to the International Classification of Diseases and Related Health Problems, 10th revision, Australian Modification (ICD‐10‐AM) and the Australian Classification of Health Interventions (ACHI). The RBDM and COD‐URF record deaths of NSW residents and provide date of death. The CCR records cancer diagnoses, and demographic and clinical information. CCR and SCOPE data were probabilistically linked by the NSW Centre for Health Record Linkage (CHeReL). Ethical approval was obtained from the NSW Population and Health Services Research Ethics Committee (2018HRE0201).

### 2.2 Case Definition

Individuals with pancreatic or periampullary cancer were identified from hospital and/or cancer registry data using the ICD‐10‐AM codes C24.0, C24.1, C24.9, C25 (Table 1). All histopathological types of pancreatic cancer were included. Though recent evidence has suggested periampullary cancer subtypes may experience different trajectories [21, 22], our approach is consistent with previous studies that group tumours of the ampullary complex, distal common bile duct and pancreatic head, given the difficulty of distinguishing these preoperatively [23, 24]. The index record was defined as the earliest record of pancreatic and periampullary cancer in cancer registry or hospitalisation records. The cancer registry defines the date of diagnosis as the earliest diagnostic or treatment episode notified to the registry, which may differ from the actual clinical date of diagnosis. A 2‐year exclusion criteria (1 January 2007 to 31 December 2008) was used to ensure the most accurate identification of the index record. Individuals with an index record after 31 December 2018 were excluded, allowing for 3‐year follow‐up data.

**TABLE 1:** Diagnosis and procedure codes for pancreatic and periampullary cancer and pancreatectomy.

**ICD‐10‐AM** ^a^ **primary diagnosis codes**	
C24.0	Malignant neoplasm of extrahepatic bile duct
C24.1	Malignant neoplasm of ampulla of Vater
C24.9	Malignant neoplasm of biliary tract, unspecified
C25	Malignant neoplasm of pancreas
**Australian Classification of Health Intervention (ACHI) procedure codes**	
30593‐00	Pancreatectomy
30593‐01	Pancreatectomy with splenectomy
30583‐00	Distal pancreatectomy
30584‐00	Pancreaticoduodenectomy with or without formation of stoma

^a^ The International Statistical Classification of Diseases and Related Health Problems, Tenth Revision, Australian Modification.

### 2.3 Outcome Measures

Primary outcomes were 1‐year, 3‐year and 5‐year all‐cause survival for [1] individuals with pancreatic or periampullary cancer, and [2] individuals with pancreatic or periampullary cancer who underwent pancreatectomy. Pancreatectomy was identified using ACHI codes (30593‐00, 30593‐01, 30583‐00, 30584‐00) (Table 1) and was separated into pancreaticoduodenectomy and non‐pancreaticoduodenectomy (total pancreatectomy, distal pancreatectomy with or without splenectomy) procedures. Individuals were classified as non‐resected if pancreatectomy was not recorded, occurred more than 365 days following (*n* = 32 (0.24%)) or more than 30 days before (*n* = 38 (0.28%)) the index record date. Survival time was calculated from index date of diagnosis to date of death, and time to resection from index date of diagnosis to index date of resection. We had follow‐up for 1‐ and 3‐year survival for all patients and restricted the cohort to patients admitted before 1 January 2017 for 5‐year survival.

### 2.4 Covariates

Year was defined as the year of the index date of diagnosis. Demographic characteristics included: sex, age, Charlson Comorbidity Index (CCI) score and extent of spread. Age at index record was categorised as 50–59, 60–69, 70–79 and ≥ 80 years. CCI score was calculated using the modified Quan coding algorithm [25], excluding pancreatic and periampullary cancer, and categorised into 0, 1–2, ≥ 3. CCI score calculation included a 2‐year lookback period to maximise identification of comorbidities. The lookback period considered all information captured in hospitalisation data in the 2 years preceding the index date. Extent of spread data was sourced from the CCR and refers to highest degree of cancer spread within 4 months of cancer diagnosis. Extent of spread was categorised into metastatic, non‐metastatic, unknown or missing groups. Metastatic, non‐metastatic and unknown classifications were derived directly from the CCR, whereas missing represented cases identified from the hospital data but absent from the CCR. Time to resection was categorised into < 30 days, 31–180 days and 181–365 days. Chemotherapy was recorded in APDC data alone and did not capture day admissions for chemotherapy. Histopathological data was not available in our dataset.

Hospital covariates included surgical volume and peer group. Volume was defined as the number of pancreatectomies for all indications at an institution in a year. We used median (six resections) and 75th percentile (16 resections) volumes to define low volume (≤ 5), medium volume [6, 7, 8, 9, 10, 11, 12, 13, 14, 15] and high volume hospitals (≥ 16) consistent with previous population‐based studies on pancreatic resection, which use the upper quartile of volume to define high volume centres [26, 27]. Peer group was classified using NSW health definitions [28], and was grouped as Private, Principal Referral (Public) (peer group A1: volume of greater than 35 000 separations and offering highly specialised services) or Major (Public) (peer groups B1, B2: volume of greater than 10 000 separations).

### 2.5 Statistical Analysis

Descriptive statistics were used to highlight demographic characteristics and survival for the whole cohort, and those who did or did not undergo pancreatectomy, and were reported as frequencies with percentages and medians with interquartile ranges (IQR). Age‐standardised incidence and pancreatectomy rates for individuals aged 50 years and older were calculated using NSW mid‐year population estimates, and directly age‐standardised to the Australian Bureau of Statistics 2001 Australian standard population using 10‐year age groups. Temporal trends in rates were calculated using negative binomial regression to account for over‐dispersion and expressed as percent annual change (PAC) with 95% confidence intervals (CIs).

Temporal trends in survival at 1‐ and 3‐years were reported for the whole pancreatic cancer and resected cohorts, and 5‐year survival trends for those diagnosed between 1 January 2009 and 31 December 2016. Kaplan–Meyer curves were used to demonstrate differences in survival. Cox's proportional hazard models were used to assess if temporal trends in 1‐, 3‐ and 5‐year survival trends remained significant after adjusting for the influence of patient (age, sex, Charlson Comorbidity Score, extent of spread, resection) and hospital‐level factors (surgical volume, peer group) and were expressed as adjusted Hazard Ratios (aHR) with 95% CIs. We assessed the proportionality assumption across all our models. Exploration of Schoenfeld residuals and visual inspection of Kaplan–Meier curves confirmed that the proportionate hazards assumption was not violated. To assess the impact of the high degree of missing information for extent of spread, we performed complete case analysis where patients with either unknown or missing extent of spread were excluded. Analyses were conducted using SAS Enterprise Guide v8.3 [29] and R Statistical Software (v4.4.2) [30] with use of *survival* (v3.8–3) [31] and *survminer* (v0.5.0) [32] packages.

## 3 Results

### 3.1 Cohort Characteristics

**FIGURE 1:**
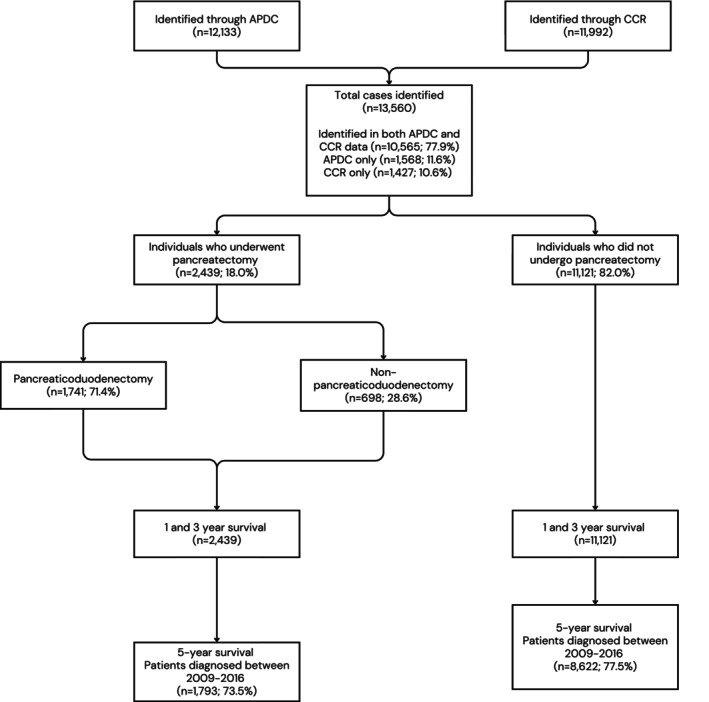
Consort diagram of identification and selection of patients aged 50 years and older, diagnosed with or admitted to hospital in New South Wales (NSW), Australia, for a diagnosis of pancreatic or periampullary cancer, linked NSW hospital (NSW Admitted Patients Data Collection (APDC)) and cancer registry (NSW Central Cancer Registry (CCR)) data, 2009–2018 (*n* = 13 560).

Across the study period, 13 560 individuals aged 50 years and older had a new diagnosis of pancreatic or periampullary cancer. Of these, 10 565 (77.9%) were identified from both hospitalisation and cancer registry data, 1427 (10.6%) individuals were identified by cancer registry data alone, and 1568 (11.6%) individuals were identified by hospitalisation data alone (Figure 1). Median age (IQR) at diagnosis was 73 (65–81) years, and 41% had a CCI score of three or more (Table 2). A total of 2439 individuals (18%) underwent pancreatectomy. The resected cohort was younger (median 68 years (IQR 61–74) vs. median 75 (IQR 66–83) years), with a higher proportion of individuals with no recorded comorbidities (50.4% vs. 38.8%) and a lower proportion of individuals with metastatic disease within 4 months of diagnosis than the non‐resected cohort (9.2% vs. 44.1%). Median time to resection (IQR) was 16 days (0–43). Chemotherapy was recorded in 15.9% of the whole cohort and 28% of the surgical cohort. This proportion is lower than previously reported, reflecting the limited capture of systemic therapy within this dataset.

**TABLE 2:** Characteristics of patients aged 50 years and older, diagnosed with or admitted to hospital in New South Wales (NSW), Australia, for a diagnosis of pancreatic or periampullary cancer, linked NSW hospital and cancer registry data, 2009–2018 (*n* = 13 560).

	Total cohort frequency (%) (*n* = 13 560)	Resected cohort frequency (%) (*n* = 2439)	Non‐resected cohort frequency (%) (*n* = 11 121)
Number of cases in both APDC^a^ and CCR^b^	10 565 (77.9)	2168 (88.9)	8397 (75.5)
Number of cases only in APDC	1568 (11.6)	200 (8.2)	1368 (12.3)
Number of cases only in CCR	1427 (10.6)	71 (2.9)	1356 (12.3)
Age, median (IQR)	73 (65–81)	68 (61–74)	75 (66–83)
Age group (years)			
50–59	1692 (12.5)	494 (20.6)	1198 (10.8)
60–69	3520 (26.0)	925 (37.9)	2595 (23.3)
70–79	4205 (31.0)	824 (33.8)	3381 (30.4)
≥ 80	4143 (30.5)	195 (8.0)	3947 (35.5)
Male sex^c^	7114 (52.5)	1365 (56.0)	5749 (51.8)
Charlson Comorbidity Index (CCI)^d^			
0	5544 (40.9)	1230 (50.4)	4314 (38.8)
1–2	2451 (18.1)	525 (21.5)	1926 (17.3)
≥ 3	5565 (41.0)	684 (28.0)	4881 (43.9)
Extent of Spread (within 4 months of diagnosis)			
Non‐metastatic	4653 (34.3)	1918 (78.6)	2735 (24.6)
Metastatic	5134 (37.9)	225 (9.2)	4909 (44.1)
Unknown^e^	2205 (16.3)	96 (3.9)	2109 (19.0)
Missing^f^	1568 (11.6)	200 (8.2)	1368 (12.3)
Resection type			
Pancreaticoduodenectomy	1741 (12.8)	1741 (71.4)	n/a
Non‐pancreaticoduodenectomy procedures	698 (5.1)	698 (28.6)	n/a
Chemotherapy^g^	1926 (15.9)	565 (23.9)	1361 (13.9)
Survival^h^			
1‐year	4824 (35.6)	1974 (80.9)	2850 (25.6)
3‐year	2499 (18.4)	1254 (51.4)	1245 (11.2)
5‐year^i^	1529 (14.7)	724 (40.4)	805 (9.3)

^a^ APDC, NSW Admitted Patient Data Collection.

^b^ CCR, NSW Central Cancer Registry.

^c^ Values missing (*n* = 14).

^d^ Charlson Comorbidity Index (CCI) score.

^e^ Recorded as unknown in CCR (New South Wales Central Cancer Registry) data.

^f^ CCR data not available.

^g^ CCR data not available for chemotherapy: *n* = 1427 missing (total cohort), *n* = 71 missing (resected cohort), *n* = 1356 missing (non‐resected cohort).

^h^ (*n* = 4) values missing.

^i^ Patients diagnosed in 2017–2018 were not included due to lack of 5‐year follow‐up data. Total cohort 2009–16: (*n* = 10 415) analysed, Resected cohort 2009–16: (*n* = 1793) analysed, Non‐resected cohort, 2009–16: (*n* = 8622) analysed.

### 3.2 Temporal Trends

**FIGURE 2:**
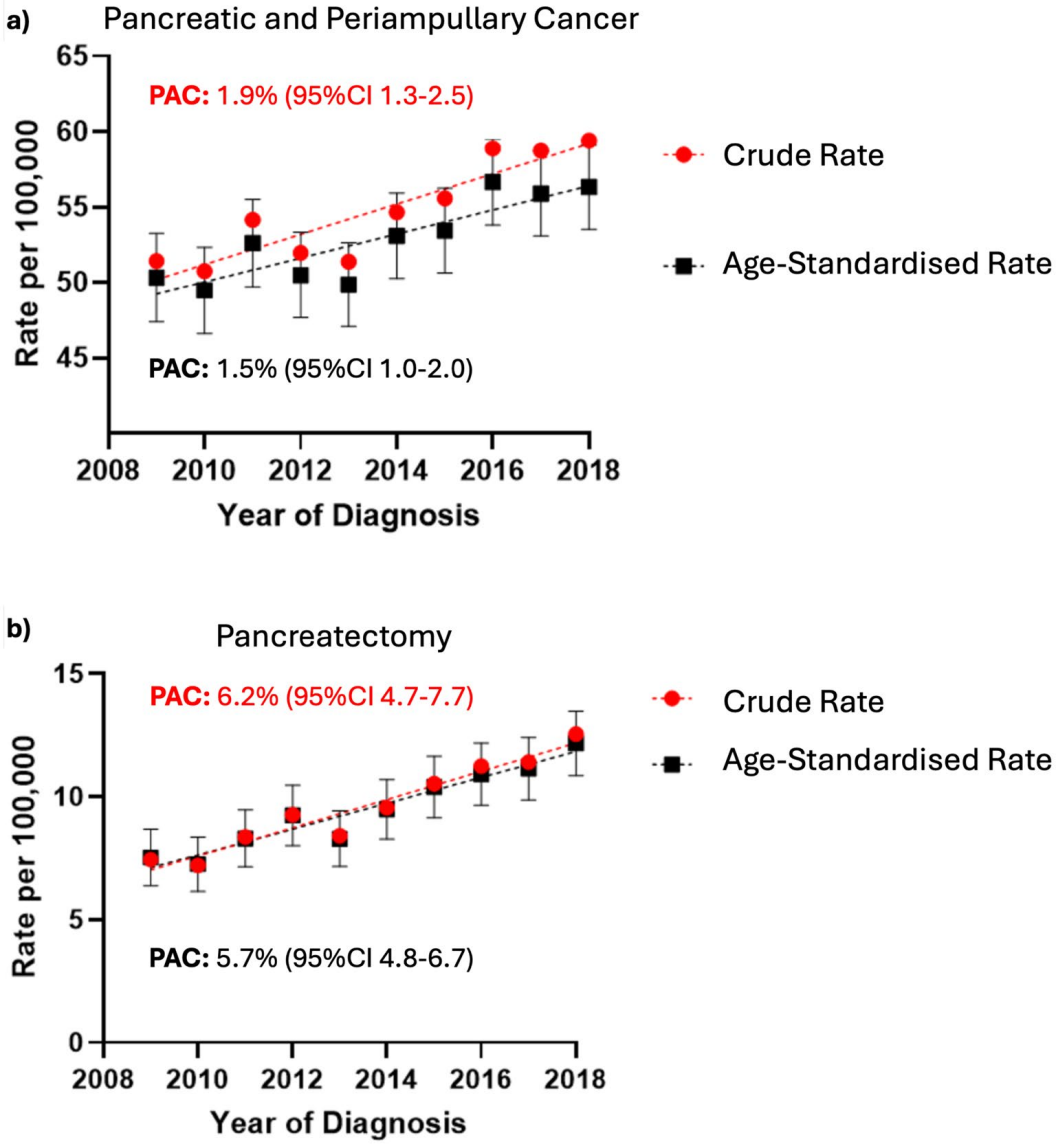
(a and b) Rates of (a) pancreatic and periampullary cancer and (b) pancreatectomy, with Percent Annual Change (PAC), of patients aged 50 years and older diagnosed with or admitted to hospital in New South Wales (NSW), Australia, for a diagnosis of pancreatic or periampullary cancer, linked NSW hospital and cancer registry data, 2009–2018 (*n* = 13 560).

In NSW, the number of pancreatic and periampullary cancer diagnoses per year in people aged 50 years and older increased from 1148 in 2009 to 1591 in 2018, an average percent annual increase of 3.9% (95% CI 3.3–4.5). Over the same period, age‐standardised rates for pancreatic and periampullary cancer in individuals aged 50 years and older increased 1.5% (95% CI 1.0–2.0) per year from 50.3/100 000 population (95% CI 47.4–53.3) to 56.3/100 000 population (95% CI 53.5–56.3) (Figure 2a). The number of pancreatectomies and the proportion of patients undergoing pancreatectomy increased from 167 (14.5%) in 2009 to 341 (21.4%) in 2018, representing an average annual increase of 8.3% (95% CI 6.8–9.9) in the number of pancreatectomies each year. Age‐standardised pancreatectomy rates for individuals aged 50 years and older increased by 5.7% (95% CI 4.8–6.7) annually, from 7.5/100 000 population (95% CI 6.4–8.7) in 2009 to 12.2/100 000 population (95% CI 10.9–13.5) in 2018 (Figure 2b). For individuals aged 70–79, age‐specific rates for pancreatectomy increased from 13.7 to 23.6 per 100 000, reflecting an average annual increase of 6.9% (95% CI 4.3–9.5). The proportion of patients recorded receiving chemotherapy increased from 10.0% to 18.5% (2009–2018).

The proportion of individuals undergoing resection decreased with increasing age, but across the duration of the study period, proportions of individuals undergoing resection increased across all age‐groups. Increases in resection rates were observed in individuals aged 60–69 (21.0%–28.4%) and 70–79 (15.7%–24.3%) (2009–2018) (Table 3). Across the study period, most procedures were pancreaticoduodenectomies (71.4%), however, the proportion of procedures represented by pancreaticoduodenectomies decreased over time from 80.2% (2009) to 65.4% (2018), with an increase in non‐pancreaticoduodenectomy resections over that time (Table 3). The proportion of patients undergoing resection who were recorded as receiving chemotherapy remained consistent between 2009–2018 (25.0% to 23.3%).

**TABLE 3:** Temporal trends in pancreatectomy for patients aged 50 years and older diagnosed with or admitted to hospital in New South Wales (NSW), Australia, for a diagnosis of pancreatic or periampullary cancer, linked NSW hospital and cancer registry data, 2009–2018 (*n* = 2439).

	2009 (*n* = 167/1148)^a^	2010 (*n* = 167/1163)^a^	2011 (*n* = 201/1272)^a^	2012 (*n* = 226/1249)^a^	2013 (*n* = 216/1263)^a^	2014 (*n* = 246/1372)^a^	2015 (*n* = 271/1419)^a^	2016 (*n* = 299/1529)^a^	2017 (*n* = 305/1550)^a^	2018 (*n* = 341/1591)^a^
Resection rate	14.5%	14.4%	15.8%	18.1%	17.1%	17.9%	19.1%	19.6%	19.7%	21.4%
Resection type (*n*, %)										
Pancreaticoduodenectomy	134 (80.2)	125 (74.9)	155 (77.1)	164 (72.6)	166 (76.9)	163 (66.3)	192 (70.9)	218 (72.9)	201 (65.9)	223 (65.4)
Non‐pancreaticoduodenectomy procedures	33 (19.8)	42 (25.1)	46 (22.9)	62 (27.4)	50 (23.1)	83 (33.7)	79 (29.1)	81 (27.1)	104 (34.1)	118 (34.6)
Resection rate by age group (*n*, %)										
50–59	42 (28.8)	36 (25.5)	42 (26.8)	53 (31.0)	35 (22.4)	47 (27.0)	60 (35.1)	60 (28.3)	56 (31.3)	63 (34.1)
60–69	56 (21.0)	54 (19.6)	84 (25.3)	92 (28.1)	89 (27.3)	90 (24.3)	103 (26.3)	120 (30.8)	111 (28.0)	126 (28.4)
70–79	57 (15.7)	58 (16.5)	56 (15.2)	69 (19.0)	68 (17.8)	89 (20.7)	88 (20.6)	93 (19.5)	116 (22.8)	130 (24.3)
≥ 80	12 (6.3)	19 (9.3)	19 (9.8)	12 (6.6)	24 (12.6)	20 (10.1)	20 (10.0)	26 (12.1)	22 (9.4)	22 (11.1)
Resection rate by Charlson Comorbidity Index Score (*n*, %)										
0	78 (17.4)	97 (18.8)	112 (18.2)	137 (23.4)	113 (21.6)	117 (20.9)	124 (23.3)	139 (24.4)	144 (25.0)	169 (27.4)
1–2	31 (15.7)	32 (17.8)	30 (17.0)	40 (20.4)	48 (19.1)	63 (24.1)	64 (23.6)	61 (21.6)	81 (23.3)	75 (26.3)
≥ 3	58 (11.6)	38 (8.2)	59 (12.3)	49 (10.5)	55 (11.3)	66 (12.0)	83 (13.5)	99 (14.6)	80 (12.8)	97 (14.1)
Time to resection (*n*, %)										
0–30 days	123 (72.8)	109 (65.3)	143 (71.1)	150 (63.4)	141 (65.3)	155 (63.0)	184 (67.9)	200 (66.9)	178 (58.4)	202 (59.2)
31–180	42 (25.2)	53 (31.7)	55 (27.4)	69 (30.5)	70 (32.4)	76 (30.9)	74 (27.3)	83 (27.8)	100 (32.8)	121 (35.5)
181–365	^f^	^f^	^f^	7 (3.1)	^f^	15 (6.1)	13 (4.8)	16 (5.5)	27 (8.9)	18 (5.3)
Extent of Spread within 4 months of diagnosis (*n*, %)										
Local	126 (75.5)	136 (81.4)	156 (77.6)	177 (78.3)	171 (79.2)	201 (81.7)	210 (77.5)	241 (80.6)	228 (74.8)	272 (70.8)
Metastatic	26 (15.6)	15 (9.0)	21 (10.5)	26 (11.5)	17 (7.9)	17 (6.9)	20 (7.4)	27 (9.0)	31 (10.2)	25 (7.3)
Unknown	^f^	^f^	^f^	7 (3.1)	7 (3.24)	11 (4.5)	14 (5.2)	12 (4.0)	18 (5.9)	17 (5.0)
Missing	11 (6.6)	15 (9.0)	19 (9.5)	16 (7.1)	21 (9.7)	17 (6.9)	27 (10.0)	19 (6.4)	28 (9.2)	27 (7.9)
Hospital volume (*n*, %)										
High volume (≥ 16)	94 (56.3)	97 (58.1)	122 (60.7)	133 (58.9)	130 (60.2)	170 (69.1)	200 (73.8)	243 (81.3)	239 (78.4)	274 (80.4)
Medium volume (6–15)	49 (29.3)	50 (29.9)	53 (26.4)	70 (31.0)	70 (32.4)	62 (25.2)	52 (19.2)	43 (14.4)	46 (15.1)	52 (15.3)
Low volume (≤ 5)	24 (14.4)	20 (12.0)	26 (12.9)	23 (10.2)	23 (7.4)	14 (5.7)	19 (7.0)	13 (4.4)	20 (6.6)	15 (4.4)
Peer group^b^ (*n*, %)										
Principal referral^c^	100 (59.9)	109 (65.3)	118 (58.7)	113 (50.0)	130 (60.2)	135 (54.9)	130 (48.2)	162 (54.2)	164 (53.8)	190 (55.7)
Major^d^	17 (10.2)	17 (10.2)	17 (8.5)	29 (12.8)	22 (10.2)	15 (6.1)	21 (7.8)	12 (4.0)	17 (5.6)	19 (5.6)
Private	50 (29.9)	41 (24.6)	66 (32.8)	84 (37.2)	64 (29.6)	96 (39.0)	119 (44.1)	125 (41.8)	124 (40.7)	132 (38.7)
Chemotherapy^e^	41 (25.0)	29 (17.9)	40 (20.6)	53 (24.8)	32 (15.3)	59 (24.8)	79 (29.9)	85 (29.2)	70 (23.3)	77 (23.3)

^a^ The number of patients who underwent resection is shown as the numerator, while the denominator represents the total cohort of patients diagnosed with pancreatic or periampullary cancer each year.

^b^ (*n* = 1) missing values.

^c^ Principal referral: volume of greater than 35 000 separations and offering highly specialised services.

^d^ Major: volume of greater than 10 000 separations.

^e^ (*n* = 71) missing values.

^f^ Cell sizes < 6 have been suppressed due to ethical requirements.

Between 2009–2018, proportions of individuals without previously recorded comorbidities undergoing pancreatic resection increased from 17.4% to 27.4%. Similarly, the proportion of individuals with one to two comorbidities undergoing resection increased from 15.7% to 26.3%. The proportion of individuals with a CCI score ≥ 3 undergoing resection increased from 11.6% to 14.1% over the same period. Time to resection increased between 2009–2018, with the percentage of individuals undergoing resection within 30 days of diagnosis decreasing from 72.8% to 59.2%. Concurrently, proportions of individuals undergoing resection between 31–180 days increased from 25.2% to 35.5%. Increasing adoption of neoadjuvant chemotherapy for select populations may contribute to increased time to surgery. Rates of metastatic extent of spread up to 4 months following diagnosis in individuals who underwent resection decreased from 15.6% to 7.3%. This likely represents individuals who underwent resection and were later found to have metastatic disease, and may represent early treatment failure.

Most (69.8%) pancreatectomies were undertaken in high volume centres. The number of high volume and medium volume hospitals increased (9 to 15 and 6 to 10, respectively), whilst the number of low volume centres decreased (17–12). The proportion of resections in high volume centres increased from 56.3% to 80.4%, whilst proportions of resections in medium (29.3%–15.3%) and low volume centres decreased (14.4%–4.4%). The majority (55.4%) of pancreatectomies were conducted in principal referral centres, followed by private hospitals (37.0%), and the percentage of pancreatectomies conducted in private hospitals increased from 29.9% to 38.7% over the study period.

## 4 Survival

**FIGURE 3:**
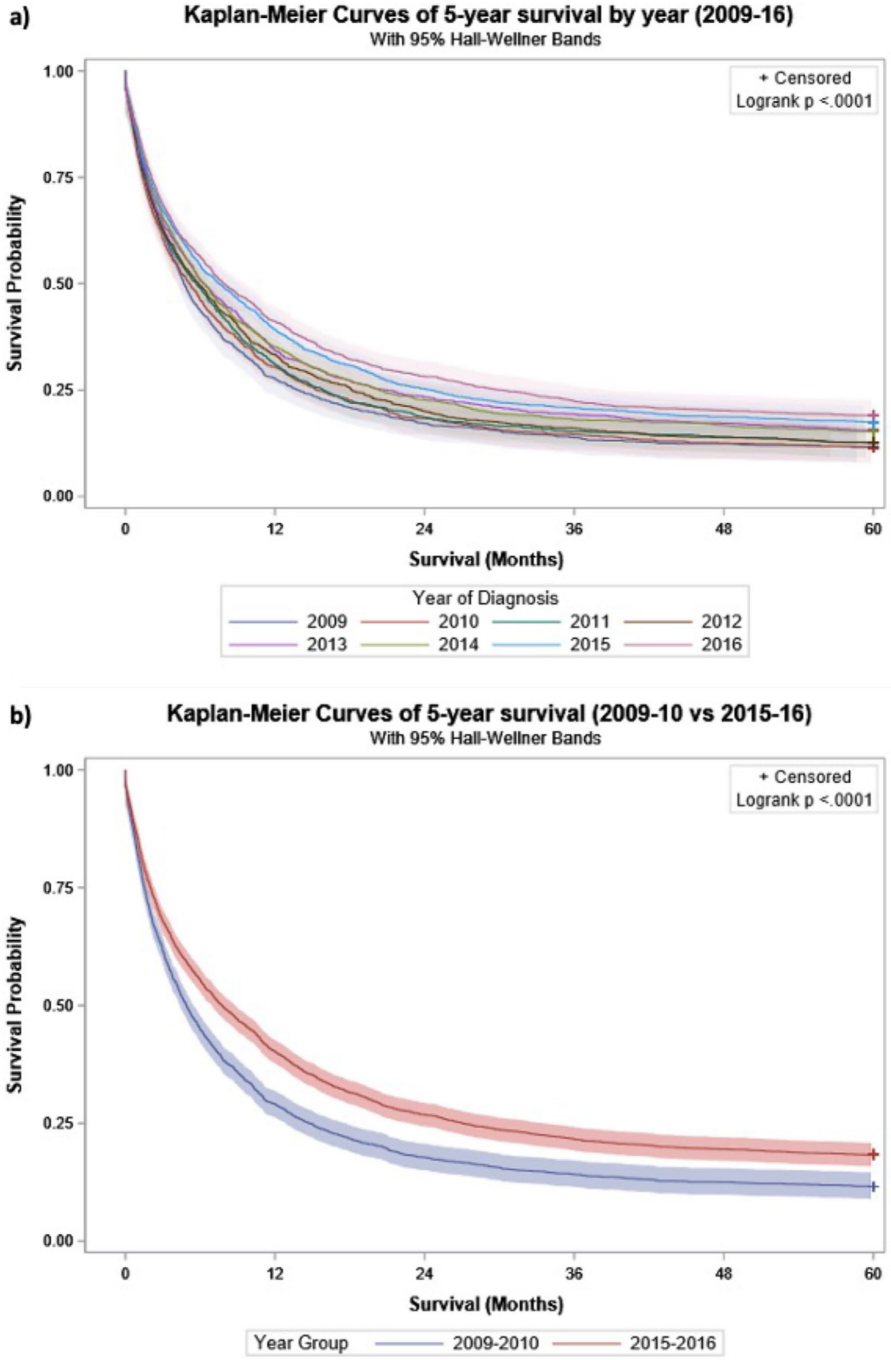
(a and b) Kaplan–Meier curves (with 95% Hall‐Wellner bands) comparing 5‐year survival of patients aged 50 years and older diagnosed with or admitted to hospital in New South Wales (NSW), Australia, for a diagnosis of pancreatic or periampullary cancer, linked NSW hospital and cancer registry data, 2009–2016; (a) by year (*n* = 10 415); (b) between 2009–10 (*n* = 2311) and 2015–2016 (*n* = 2949).

Table 4 highlights that 1‐, 3‐ and 5‐year survival rates improved over time across all levels of extent of spread. One‐year survival increased from 27.7% in 2009 to 39.7% in 2018, with increases also observed among persons diagnosed with non‐metastatic cancer (46.6% to 58.8%), and metastatic cancer (13.0% to 18.8%). Whole cohort 3‐year survival increased from 13.8% (2009) to 20.9% (2018); non‐metastatic cancer (21.0% to 32.3%) and metastatic cancer (4.3% to 5.5%). Whole cohort 5‐year survival increased from 11.6% to 19% (2009–2016); non‐metastatic disease (17.5% to 26.2%) and metastatic cancer (3.7% to 5.8%). The Hall‐Wellner confidence bands on the Kaplan–Meier curve demonstrate improved survival between periods 2009–2010 and 2015–2016 (*p* < 0.0001) (Figure 3). After adjusting for age, sex, CCI class, resection and extent of spread, survival improvements across years remain significant. Diagnosis in 2018 was associated with an 18% (aHR 0.82, 95% CI 0.74–0.89) reduced risk of 1‐year mortality and 16% (aHR 0.84, 95% CI 0.77–0.91) reduction in 3‐year mortality compared to diagnosis in 2009 (Table 5). Likewise, diagnosis in 2016 was associated with a 21% (aHR 0.79, 95% CI 0.73–0.86) reduced risk of 5‐year mortality compared to diagnosis in 2009.

**TABLE 4:** Temporal trends in 1‐, 3‐ and 5‐year survival by extent of spread within 4 months of diagnosis and surgery type, expressed as a percentage of total diagnoses each year, of patients aged 50 years and older diagnosed with or admitted to hospital in New South Wales (NSW), Australia, for a diagnosis of pancreatic or periampullary cancer, linked NSW hospital and cancer registry data, 2009–2018 (*n* = 13 560).

	2009	2010	2011	2012	2013	2014	2015	2016	2017^a^	2018^a^
Whole cohort (*n*, %)										
Number of diagnoses	1148	1163	1272^b^	1249^c^	1263	1372	1419^c^	1529	1550	1591
1‐year survival	318 (27.7)	354 (30.4)	394 (31.0)	416 (33.3)	433 (34.3)	482 (35.1)	558 (39.3)	629 (41.1)	608 (39.2)	632 (39.7)
3‐year survival	158 (13.8)	168 (14.5)	193 (15.2)	200 (16.0)	243 (19.2)	247 (18.0)	296 (20.9)	343 (22.4)	318 (20.5)	333 (20.9)
5‐year survival	133 (11.6)	133 (11.4)	160 (12.6)	159 (12.7)	197 (15.6)	209 (15.2)	248 (17.5)	290 (19.0)	n/a^a^	n/a^a^
Non‐metastatic cancer (*n*, %)										
Number of diagnoses	348	393	423^c^	405	413	469	472	576	559	595
1‐year survival	162 (46.6)	202 (51.4)	211 (49.9)	240 (59.3)	211 (51.1)	254 (54.2)	264 (55.9)	347 (60.2)	314 (56.2)	350 (58.8)
3‐year survival	73 (21.0)	93 (23.7)	106 (25.1)	113 (27.9)	117 (28.3)	126 (26.9)	143 (30.3)	191 (33.2)	161 (28.8)	192 (32.3)
5‐year survival	61 (17.5)	76 (19.3)	83 (19.6)	91 (22.5)	93 (22.5)	104 (22.2)	116 (24.6)	151 (26.2)	n/a^a^	n/a^a^
Metastatic cancer (*n*, %)										
Number of diagnoses	484	472	500^c^	510	486	507	519	517	572	565
1‐year survival	63 (13.0)	57 (12.1)	66 (13.2)	74 (14.5)	75 (15.4)	91 (18.0)	113 (21.8)	111 (21.5)	113 (19.8)	106 (18.8)
3‐year survival	21 (4.3)	22 (4.7)	21 (4.2)	28 (5.5)	30 (6.2)	36 (7.1)	36 (6.9)	36 (7.0)	45 (7.9)	31 (5.5)
5‐year survival	18 (3.7)	19 (4.0)	20 (4.0)	14 (2.8)	26 (5.4)	31 (6.1)	30 (5.8)	30 (5.8)	n/a^a^	n/a^a^
Pancreatectomy cohort^d^										
Whole pancreatectomy cohort (*n*, %)										
Individuals undergoing resection	167	167	201	226	216	246	271	299	305	341
1‐year survival	120 (71.9)	119 (71.3)	150 (74.6)	185 (81.9)	176 (81.5)	191 (77.6)	226 (83.4)	259 (86.6)	255 (83.6)	293 (85.9)
3‐year survival	71 (42.5)	68 (40.7)	92 (45.8)	106 (46.9)	117 (54.2)	113 (45.9)	151 (55.7)	174 (58.2)	167 (54.8)	195 (57.2)
5‐year survival	60 (35.9)	55 (32.9)	71 (35.3)	87 (38.5)	98 (45.4)	91 (37.0)	124 (45.8)	138 (46.2)	n/a^a^	n/a^a^
Pancreatoduodenectomy (*n*, %)										
Individuals undergoing resection	134	125	155	164	166	163	192	218	201	223
1‐year survival	95 (70.9)	88 (70.4)	114 (73.6)	130 (79.3)	130 (78.3)	123 (75.5)	153 (79.7)	186 (85.3)	166 (82.6)	187 (83.9)
3‐year survival	56 (41.8)	46 (36.8)	68 (43.9)	69 (42.1)	85 (51.2)	62 (38.0)	88 (45.8)	114 (52.3)	101 (50.3)	114 (51.1)
5‐year survival	46 (34.3)	35 (28.0)	50 (32.3)	54 (32.9)	70 (42.2)	49 (30.1)	67 (34.9)	81 (37.2)	n/a^a^	n/a^a^
Non‐Pancreatoduodenectomy (*n*, %)										
Individuals undergoing resection	33	42	46	62	50	83	79	81	104	118
1‐year survival	25 (75.8)	31 (73.8)	36 (78.3)	55 (88.7)	46 (92.0)	68 (81.9)	73 (92.4)	73 (90.1)	89 (85.6)	106 (89.8)
3‐year survival	15 (45.5)	22 (52.4)	24 (52.2)	37 (59.7)	32 (64.0)	51 (61.5)	63 (79.8)	60 (74.1)	66 (63.5)	81 (68.6)
5‐year survival	14 (42.2)	20 (47.6)	21 (45.7)	33 (53.2)	28 (56.0)	42 (50.6)	57 (72.2)	57 (70.4)	n/a^a^	n/a^a^
Non‐metastatic cancer (*n*, %)										
Individuals undergoing resection	126	136	156	177	171	201	210	241	228	272
1‐year survival	89 (70.6)	102 (75.0)	121 (77.6)	149 (84.2)	140 (81.9)	155 (77.1)	176 (83.8)	213 (88.4)	195 (85.5)	235 (86.4)
3‐year survival	51 (40.5)	57 (41.9)	71 (45.5)	83 (46.9)	92 (53.8)	87 (43.3)	114 (54.3)	140 (58.1)	124 (54.4)	151 (55.5)
5‐year survival	42 (33.3)	44 (32.4)	52 (33.3)	69 (39.0)	76 (44.4)	70 (34.8)	91 (44.3)	110 (45.6)	n/a^a^	n/a^a^
Metastatic cancer (*n*, %)										
Individuals undergoing resection	26	15	21	26	17	17	20	27	31	25
1‐year survival	18 (69.2)	6 (40.0)	8 (38.1)	17 (65.4)	10 (58.8)	12 (70.6)	16 (80.0)	16 (59.3)	18 (58.1)	16 (64)
3‐year survival	7 (26.9)	^e^	^e^	9 (34.5)	^e^	8 (47.1)	8 (40.0)	7 (25.9)	9 (29.0)	11 (44.0)
5‐year survival	^e^	^e^	^e^	^e^	^e^	7 (41.2)	7 (35.0)	^e^	n/a^a^	n/a^a^

^a^ 5‐year survival data not available for 2017–2018.

^b^ (*n* = 2) missing survival data.

^c^ (*n* = 1) missing survival data.

^d^ (*n* = 2439) analysed.

^e^ Cell sizes < 6 have been suppressed due to ethical requirements.

**TABLE 5:** Cox's proportional hazard ratios with 95% Confidence Intervals (aHR (95% CI)), adjusted for year of diagnosis, age, sex, Charlson Comorbidity Score, extent of spread in both cohorts, with additional adjustment for pancreatectomy for the whole cohort (*n* = 13 560), and additional adjustment for hospital volume and hospital peer group for the pancreatectomy cohort (*n* = 2439), for patients aged 50 years and older diagnosed with or admitted to hospital in New South Wales (NSW), Australia, for a diagnosis of pancreatic or periampullary cancer, linked NSW hospital and cancer registry data, 2009–2018.

Characteristic	All cohort (*n* = 13 560)			Pancreatectomy (*n* = 2439)		
	1‐year^a^	3‐year^a^	5‐year^b^	1‐year^c^	3‐year^c^	5‐year^d^
Year						
2009	1.00	1.00	1.00	1.00	1.00	1.00
2010	0.97 (0.88–1.07)	0.97 (0.89–1.06)	0.98 (0.9–1.07)	1.09 (0.73–1.63)	1.11 (0.84–1.48)	1.13 (0.87–1.48)
2011	0.96 (0.87–1.05)	0.96 (0.88–1.05)	0.96 (0.89–1.05)	1.05 (0.71–1.57)	1.02 (0.77–1.34)	1.07 (0.83–1.38)
2012	0.93 (0.85–1.03)	0.95 (0.87–1.04)	0.97 (0.89–1.05)	0.69 (0.45–1.06)	0.88 (0.67–1.16)	0.91 (0.71–1.18)
2013	0.88 (0.8–0.97)	0.86 (0.79–0.94)	0.87 (0.8–0.95)	0.68 (0.44–1.04)	0.74 (0.56–0.98)	0.77 (0.59–1)
2014	0.9 (0.82–0.99)	0.9 (0.83–0.98)	0.9 (0.83–0.98)	0.87 (0.59–1.29)	0.95 (0.73–1.24)	0.98 (0.76–1.25)
2015	0.77 (0.7–0.84)	0.79 (0.73–0.86)	0.8 (0.73–0.87)	0.65 (0.43–0.98)	0.71 (0.54–0.93)	0.75 (0.59–0.97)
2016	0.77 (0.7–0.84)	0.79 (0.72–0.86)	0.79 (0.73–0.86)	0.48 (0.31–0.74)	0.62 (0.48–0.81)	0.69 (0.54–0.88)
2017	0.8 (0.73–0.88)	0.81 (0.75–0.88)	n/a	0.66 (0.44–0.99)	0.75 (0.57–0.97)	n/a
2018	0.82 (0.74–0.89)	0.84 (0.77–0.91)	n/a	0.54 (0.36–0.82)	0.65 (0.5–0.84)	n/a
Age/year	1.04 (1.04–1.04)	1.03 (1.03–1.03)	1.03 (1.03–1.03)	1.04 (1.03–1.05)	1.03 (1.02–1.03)	1.03 (1.02–1.03)
Sex						
Male	1.00	1.00	1.00	1.00	1.00	1.00
Female	1.04 (0.99–1.08)	1.03 (0.99–1.06)	1.01 (0.97–1.05)	0.91 (0.76–1.09)	0.92 (0.82–1.03)	0.88 (0.77–0.99)
CCI^e^						
0	1.00	1.00	1.00	1.00	1.00	1.00
1–2	1.15 (1.08–1.23)	1.1 (1.04–1.16)	1.11 (1.05–1.18)	1.11 (0.87–1.42)	0.93 (0.8–1.09)	0.99 (0.84–1.16)
≥ 3	1.72 (1.64–1.8)	1.57 (1.51–1.64)	1.6 (1.52–1.68)	1.47 (1.2–1.82)	1.3 (1.14–1.48)	1.3 (1.13–1.5)
Extent of spread						
Non‐metastatic	1.00	1.00	1.00	1.00	1.00	1.00
Metastatic	1.98 (1.87–2.1)	1.75 (1.67–1.84)	1.76 (1.66–1.86)	2.44 (1.92–3.1)	1.9 (1.6–2.25)	1.77 (1.47–2.14)
Unknown	1.42 (1.33–1.52)	1.22 (1.15–1.3)	1.24 (1.16–1.32)	0.76 (0.42–1.35)	0.78 (0.56–1.08)	0.82 (0.57–1.17)
Missing	0.95 (0.88–1.03)	0.68 (0.63–0.73)	0.68 (0.63–0.74)	0.58 (0.38–0.9)	0.38 (0.28–0.51)	0.38 (0.28–0.51)
Pancreatectomy						
No	1.00	1.00	1.00			
Yes	0.24 (0.22–0.26)	0.36 (0.33–0.38)	0.41 (0.38–0.44)			
Hospital volume						
Low (≤ 5)				1.00	1.00	1.00
Medium (6–15)				1.05 (0.74–1.5)	1.08 (0.85–1.37)	1.11 (0.87–1.42)
High (≥ 16)				0.88 (0.62–1.25)	1.12 (0.89–1.42)	1.19 (0.94–1.51)
Hospital peer group						
Principal referral				1.00	1.00	1.00
Major				1 (0.68–1.47)	1.2 (0.93–1.55)	1.21 (0.93–1.57)
Private				0.77 (0.62–0.94)	0.87 (0.77–0.99)	0.91 (0.79–1.04)

^a^ (*n* = 18) individuals excluded from analysis due to missing values (*n* = 13 542) analysed.

^b^ Individuals diagnosed in 2017–2018 were not included due to lack of 5 year follow up. (*n* = 17) individuals excluded from analysis due to missing values, (*n* = 10 402) analysed.

^c^ (*n* = 3) individuals excluded from analysis due to missing values (*n* = 2436) analysed.

^d^ Individuals diagnosed in 2017–2018 were not included due to lack of 5 year follow up. (*n* = 3) individuals excluded from analysis due to missing values, (*n* = 1790) analysed.

^e^ Charlson Comorbidity Index (CCI) score.

**FIGURE 4:**
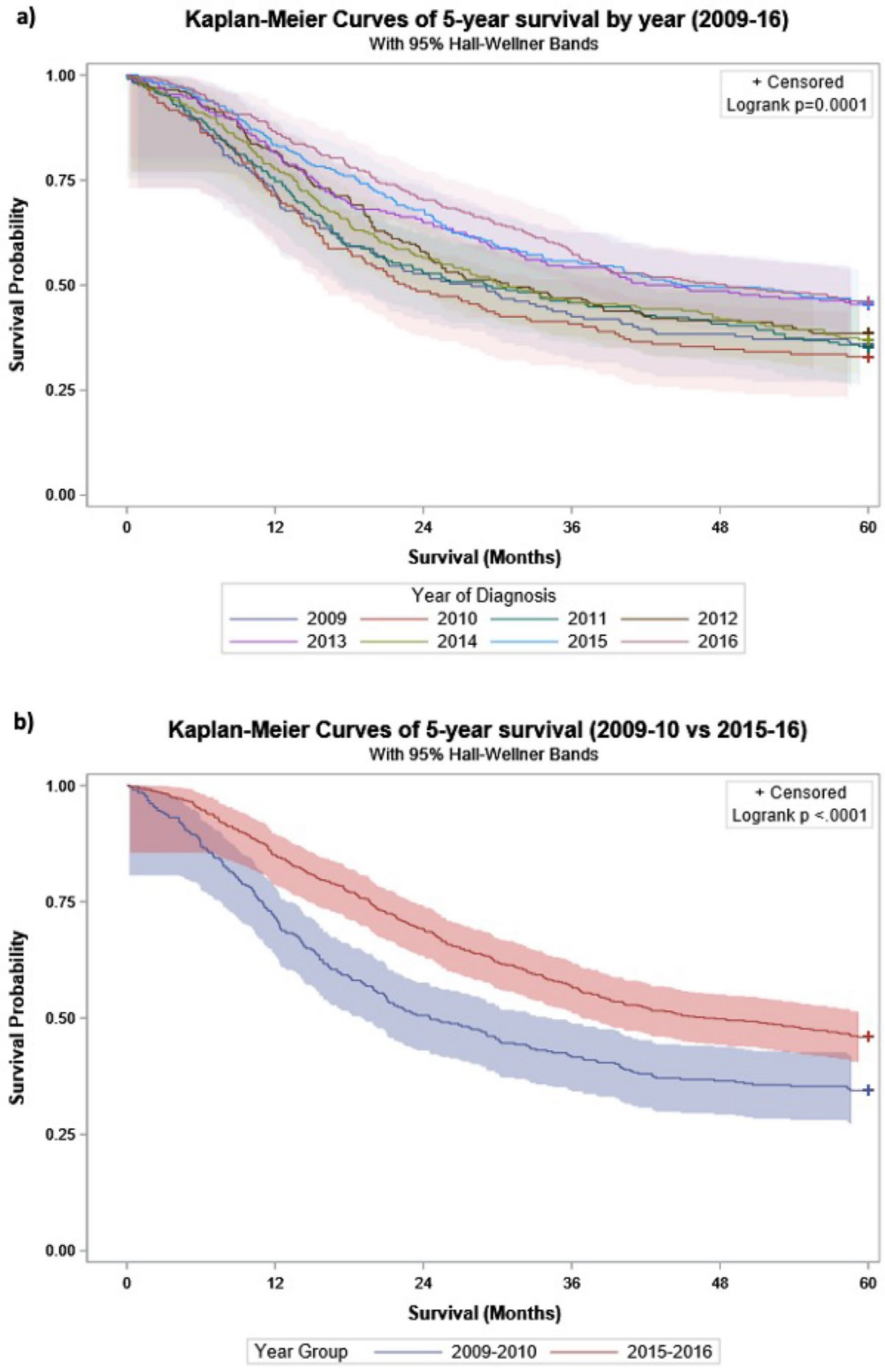
(a and b) Kaplan–Meier curves (with 95% Hall‐Wellner bands) comparing 5‐year survival of patients aged 50 years and older undergoing pancreatectomy, diagnosed with or admitted to hospital in New South Wales (NSW), Australia, for a diagnosis of pancreatic or periampullary cancer, linked NSW hospital and cancer registry data, 2009–2016; (a) by year (*n* = 1793); (b) between 2009–10 (*n* = 334) and 2015–16 (*n* = 570).

Survival rates amongst patients who underwent pancreatectomy improved at 1‐year (71.9% to 85.9%) and 3‐years (42.5% to 57.2%) from 2009 to 2018 and at 5‐years (35.9% to 46.2%) from 2009 to 2016 (Table 4). For patients undergoing pancreatoduodenectomy, 1‐year survival rose from 70.9% in 2008 to 83.9% in 2018, 3‐year survival from 41.8% to 51.1% (2008–2018), and 5‐year survival from 28.0% in 2009 to 37.2% in 2016, with the highest 5‐year survival being 42.2% (2013). In patients undergoing non‐pancreatoduodenectomy procedures, 1‐year survival increased from 75.8% in 2009 to 89.8% in 2018, 3‐year survival from 45.5% to 68.6% (2009–2018) and 5‐year survival from 42.2% in 2009 to 70.4% in 2016. Kaplan–Meier analysis (Figure 4) demonstrates significant differences in survival between years. After adjusting for age, sex, CCI class, spread, hospital volume and peer group, survival improvements across years remain significant (Table 5). Pancreatectomy patients diagnosed in 2018 compared to 2009 had a 46% and 35% lower risk of death at 1‐year (aHR 0.54, 95% CI 0.36–0.82) and 3‐years (aHR 0.65, 95% CI 0.50–0.84) respectively. There was a 31% lower risk of death at 5‐years for those diagnosed in 2016 compared to 2009 (aHR 0.69, 95% CI 0.54–0.88). Resection in a private hospital was associated with increases in 1‐ (aHR 0.77, 95% CI 0.62–0.94) and 3‐ (aHR 0.87, 95% CI 0.77–0.99) year survival, but not 5‐year survival (aHR 0.92, 95% CI 0.8–1.05). Results from the complete case analysis provided adjusted Hazard Ratios similar to the primary analysis (Table S1).

## 5 Discussion

This is the first population‐based retrospective cohort study in New South Wales to examine trends in pancreatic and periampullary cancer rates, pancreatectomy, and survival in people aged 50 years and older, utilising cancer registry and hospitalisation data to identify additional cases. Increased rates of pancreatic and periampullary cancer are observed, potentially related to population ageing, changing prevalence of risk factors, and improved diagnostic tools [1, 33]. Diverging annual crude and age‐standardised rates (Figure 2a) reflect an ageing population and increased incidence with age. Previous Australian analysis supports an increasing annual age‐standardised rate of pancreatic cancer (1995–2014) [5] and increased incidence in adults aged 50 years and older (1990–2017) [34]. We identified a median age of 73 years, comparable to other reports [3]. Given predictions about the aging population, the number of people with pancreatic and periampullary cancer is expected to continue to increase [35].

Over the study period, 5‐year survival improved and is comparable to recent international data, which notes 5‐year survival between 31.4% and 43.2% [36]. One‐, three‐ and five‐year survival in the pancreatoduodenectomy cohort compares well with previous reports. In the pancreatoduodenectomy cohort, improvements in 1‐ and 3‐year survival were more marked than increases in 5‐year survival. Improved survival over our study's duration is in concordance with prior research [34, 37]. We report improved 1‐year survival for patients with non‐metastatic disease compared to previous reports. Previous Australian analysis by stage (2012–2014) reveals 1‐year survival for non‐metastatic cancer (46%) lower than our study (55%) [38] and slightly poorer 1‐year survival in metastatic cancer (15% vs. 17%) and 3‐year survival in metastatic (3.6%) and non‐metastatic (26%) cancer than our study (6% and 28%) [38].

Both pancreatoduodenectomy and non‐pancreatoduodenectomy cohorts noted survival improvements over time. This may reflect improvements in the numbers of patients considered for pancreatectomy due to improved pre‐operative optimisation of comorbidities, increasing evidence for extended resection, efficacy of modern systemic therapy, or the widespread use of imaging leading to earlier diagnosis [17, 18, 33]. Earlier diagnosis, as well as surveillance of patients with genetic predisposition and pre‐malignancy may lead to stage migration, affecting these outcomes [33]. Among individuals who underwent resection, decreasing rates of metastatic cancer detected within 4 months of diagnosis may suggest improving surgical candidate selection and decreasing rates of early treatment failure. Our analysis included all histopathological types of pancreatic cancer, not solely pancreatic ductal adenocarcinoma. Given the comparatively better prognosis of non‐pancreatic ductal adenocarcinoma subtypes, this broader inclusion likely contributes to the higher survival reported relative to previous Australian and international literature [39]. Reported survival may also be positively influenced by the inclusion of both pancreatoduodenectomy and non‐pancreatoduodenectomy cohorts [40].

Our study spans a period of significant change in pancreatic cancer treatment and outcomes, marked by the introduction of novel chemotherapy regimens including FOLFIRINOX [41], gemcitabine‐based combinations [42], and S‐1 [43]. Increasing evidence supporting neoadjuvant therapy [14], which may make up to 60% of previously unresectable tumours amenable to resection [44], is beginning to translate to clinical practice. The increasing use of neoadjuvant therapy is likely the main driver of the increased time to resection observed and may facilitate surgery in people who would previously not be considered for operative intervention. In a study from Victoria, Australia (2016–2019), almost half of borderline resectable patients undergoing neoadjuvant chemotherapy ultimately underwent resection [45]. Despite improving neoadjuvant therapy, patients with borderline resectable disease still experience poorer outcomes than individuals with resectable disease [46, 47]. Numbers of patients undergoing chemotherapy captured in this study are less than previously reported. This likely reflects the nature of our datasets; CCR did not include chemotherapy, and SCOPE did not record chemotherapy day admissions.

Pancreatectomy rates of 18% are consistent with findings for contemporary periods [8, 19]. During the study period we demonstrate increasing pancreatic resection rates (14.5% to 21.4%), with the overall number of procedures more than doubling across the study period. Increases in procedure rates were most evident in the cohort aged 60–79 years old. Decreasing rates of pancreatoduodenectomy, and increasing non‐pancreatoduodenectomy resection rates may reflect a changing distribution of disease and higher incidental identification rates of asymptomatic pancreatic body and tail lesions due to increased rates of community imaging [48]. Decreased proportions of pancreatoduodenectomies identified over time may also positively affect survival, given that non‐pancreatoduodenectomy resections generally record better survival than pancreaticoduodenectomies [40]. This data has significant implications for surgical and oncology service provision and health service investment.

Changing guidelines which have supported increased centralisation of pancreatic resection and use of neoadjuvant therapy may contribute to observed increases in resection rates [13, 14, 16]. A previous study of pancreatectomy in NSW noted that patients in areas with low resection rates demonstrated poorer long‐term survival and suggested that low volume regions may have a degree of under‐resection [19], which centralisation may mitigate. Our data demonstrated an increase in numbers of medium and high volume centres and a move towards centralisation of services, which may contribute to increased procedure rates [49]. High volume centres demonstrate improved outcomes through better prevention and management of surgical complications, a multidisciplinary approach, and more timely recognition of the deteriorating patient [13, 14].

Empirical definitions of a high volume pancreatectomy centre were employed, consistent with previous international studies which use the upper quartile as the definition of high volume [26, 27]. Australia does not have an official definition for high volume [49], though the Cancer Institute NSW recommends a minimum volume of 6 pancreatectomies per year [15]. Burmeister et al. [50], in their Delphi process, raised the question of whether greater than 15 pancreatic cancer resections was a factor associated with quality of care, but amended the classification to 11 resections because only 3 hospitals from their previous study [51], using data from 2009–11, passed this threshold. Our data demonstrates a trend towards higher surgical volumes, with the number of hospitals performing at least 16 resections per year increasing from 9 in 2009 to 15 in 2018. We used a combination of cancer registry and hospital data to identify cases, which explains why we identified more high volume hospitals than in Burmeister et al. Additionally, for our calculation of volume, we included all pancreatic resections, not only those for cancer. Since the perioperative care of such patients is similar, inclusion of these cases more accurately represents a centre's ability to perform these procedures. Overall, we suggest improved adherence to volume recommendations, with fewer low volume centres now performing pancreatectomy in NSW [13, 14].

## 6 Strengths and Limitations

Our study provides real‐world longitudinal analysis of pancreatic and periampullary cancer and associated pancreatectomy, demonstrating significant changes over time. Using two datasets facilitated a comprehensive identification of pancreatic cancer patients. This highlights the pitfalls of using a single dataset, with over 10% of patients missed using only the CCR or hospital data.

We were unable to include analysis of people younger than 50 years due to the SCOPE dataset being purposed to explore surgical care of older patients, only recording data for patients aged 50 years and above. However, pancreatic cancer diagnoses before 50 years of age are uncommon, representing around 6% of cases [52]. Due to differences in clinical presentation and outcomes, it has been suggested that early onset pancreatic cancer may be a distinct clinical entity [52, 53, 54]. Recent research has suggested that pancreatic and periampullary cancer subtypes experience different trajectories [21, 22], however, our data was unable to distinguish well between these due to the lack of post‐operative histological data. Instead, our study aims to describe broad trends in pancreatic and periampullary cancer resection patterns. Without histological data, it is also not possible to distinguish between and analyse the changing incidence of neuroendocrine and adenocarcinoma tumours, which may influence the improving survival described. It has been suggested that neuroendocrine tumours have increased in incidence, and have correspondingly noted stage migration and survival improvements [39].

Administrative data includes inherent limitations. Use assumes that databases have complete capture of cases, and that conditions and procedures are coded accurately. Important patient factors that impact treatment and outcomes such as BMI are not routinely available. Cases identified only in CCR data may describe patients diagnosed with pancreatic and periampullary cancer who never presented to hospital. Conversely, cases identified only in hospital data suggest under‐reporting to the cancer registry, despite mandatory reporting regulations. Individuals with a time to resection of 0 days likely reflect those diagnosed as outpatients, whose diagnosis was not recorded in the registry until their admission for surgery. The cancer registry records the earliest notification of cancer to the registry as the date of diagnosis, which may not correspond exactly the with actual date of diagnosis, and is a limitation of this data. This may lead to an underestimation of time to resection, though the overall trend is preserved.

A significant number of individuals did not have extent of spread data. However, extent of spread was not a primary outcome and was included and adjusted for in analysis. We conducted a complete case analysis for ‘unknown’ and ‘missing’ extent of spread for both the total and surgical cohorts, which resulted in hazard ratios similar to those obtained in the primary analysis. As such, we were able to include all cases in our analysis.

Low rates of chemotherapy were identified; CCR did not include chemotherapy, and SCOPE did not record chemotherapy day admissions. Thus, the chemotherapy data analysed is likely to underestimate numbers of patients who received chemotherapy. Subsequently, we were also unable to analyse time to neoadjuvant therapy or identify time to curative‐intent treatment, only time to resection. Private hospital peer‐group was not recorded. Our data only includes NSW hospitalisation data, and as such may not record resections for a small number of patients who go interstate for treatment, as occurs in some regional NSW areas. We do not expect this to significantly affect our findings but may result in a slight underestimation in the number of pancreatectomies performed.

## 7 Conclusion

Between 2009–2018, rates of pancreatic and periampullary cancer in people aged 50 years and older increased significantly. Pancreatectomy rates are increasing, and survival of pancreatic cancer in NSW continues to improve and compares favourably to international results. Surgical patterns are changing, with increasing use of high volume centres, and higher proportions of multimorbid patients and individuals aged 60–69 and 70–79 years old being considered for resection. This study included all histopathological types of pancreatic cancer and both pancreaticoduodenectomy and non‐pancreaticoduodenectomy procedures. These inclusion criteria should be considered when comparing outcomes with other studies. If the observed temporal trends continue, this will have significant impacts on future healthcare utilisation for this disease.

## Author Contributions

**Joshua Mok:** conceptualisation; data curation; formal analysis; methodology; writing – original draft; writing – review and editing. **Jacqueline Close:** conceptualisation; funding acquisition; project administration; resources; supervision; writing – review and editing. **Robert Gandy:** conceptualisation; supervision; writing – review and editing. **Lara Harvey:** conceptualisation; formal analysis; funding acquisition; investigation; methodology; project administration; resources; supervision; writing – review and editing.

## Funding

The authors have nothing to report.

## Conflicts of Interest

The authors declare no conflicts of interest.

## Supporting information


**Table S1:** Complete case analysis of Cox's proportional hazard ratios with 95% Confidence Intervals (aHR (95% CI)), with individuals with unknown and missing extent of spread excluded, adjusted for year of diagnosis, age, sex, Charlson Comorbidity Score, extent of spread, with additional adjustment for pancreatectomy for the whole cohort, and hospital volume and hospital peer group for the pancreatectomy cohort, for patients aged 50 years and older diagnosed with or admitted to hospital in New South Wales (NSW), Australia for a diagnosis of pancreatic or periampullary cancer, linked NSW hospital and cancer registry data, 2009–2018 (*n* = 9774).

## Data Availability

The authors have nothing to report.
