# Supplementary material for: Temporal Trends in Pancreatic Cancer in New South Wales, Australia: A Longitudinal Population‐Based Study of Incidence, Surgery and Survival
Source: ANZ J Surg. 2026 Feb 26;96(4):912–28. doi: 10.1111/ans.70559 (PMC13129255; doi:10.1111/ans.70559)
Supplement: Supplementary file 1 — Table S1: Complete case analysis of Cox's proportional hazard ratios with 95% Confidence Intervals (aHR (95% CI)), with individuals with unknown and missing extent of spread excluded, adjusted for year of diagnosis, age, sex, Charlson Comorbidity Score, extent of spread, with additional adjustment for pancreatectomy for the whole cohort, and hospital volume and hospital peer group for the pancreatectomy cohort, for patients aged 50 years and older diagnosed with or admitted to hospital in New South Wales (NSW), Australia for a diagnosis of pancreatic or periampullary cancer, linked NSW hospital and cancer registry data, 2009–2018 (n = 9774). [file ANS-96-912-s001.docx]

**Supplementary material**

**Supplementary Table 1:** Complete case analysis of Cox’s proportional hazard ratios with 95% Confidence Intervals (aHR (95%CI)), with individuals with unknown and missing extent of spread excluded, adjusted for year of diagnosis, age, sex, Charlson Comorbidity Score, extent of spread, with additional adjustment for pancreatectomy for the whole cohort, and hospital volume and hospital peer group for the pancreatectomy cohort, for patients aged 50 years and older diagnosed with or admitted to hospital in New South Wales (NSW), Australia for a diagnosis of pancreatic or periampullary cancer, linked NSW hospital and cancer registry data, 2009-2018, (n=9,774).

|  | | **All cohort (n=9,774)** | | | **Pancreatectomy (n=2,141)** | | |  |
| --- | --- | --- | --- | --- | --- | --- | --- | --- |
| **Characteristic** | | **1-year**^†^ | **3-year**^†^ | **5-year**^‡^ | **1-year**^§^ | **3-year**^§^ | **5-year**^\|\|^ | |
| **Year** | |  |  |  |  |  |  | |
|  | 2009 | 1.00 | 1.00 | 1.00 | 1.00 | 1.00 | 1.00 | |
|  | 2010 | 1.00 (0.89-1.12) | 0.97 (0.88-1.08) | 0.97 (0.88-1.08) | 1.02 (0.67-1.56) | 1.03 (0.77-1.38) | 1.05 (0.8-1.39) | |
|  | 2011 | 0.99 (0.89-1.11) | 0.97 (0.88-1.07) | 0.98 (0.88-1.08) | 1.07 (0.71-1.61) | 0.99 (0.75-1.31) | 1.03 (0.79-1.34) | |
|  | 2012 | 0.9 (0.81-1.01) | 0.92 (0.83-1.02) | 0.94 (0.85-1.04) | 0.66 (0.42-1.02) | 0.83 (0.63-1.09) | 0.86 (0.67-1.12) | |
|  | 2013 | 0.93 (0.83-1.04) | 0.89 (0.81-0.99) | 0.9 (0.81-0.99) | 0.69 (0.45-1.07) | 0.7 (0.53-0.94) | 0.73 (0.56-0.96) | |
|  | 2014 | 0.89 (0.79-0.99) | 0.88 (0.79-0.97) | 0.88 (0.79-0.97) | 0.87 (0.58-1.31) | 0.91 (0.7-1.2) | 0.91 (0.71-1.18) | |
|  | 2015 | 0.78 (0.7-0.87) | 0.8 (0.72-0.88) | 0.81 (0.73-0.89) | 0.6 (0.39-0.93) | 0.67 (0.5-0.88) | 0.7 (0.54-0.91) | |
|  | 2016 | 0.75 (0.67-0.84) | 0.77 (0.7-0.85) | 0.78 (0.71-0.86) | 0.5 (0.32-0.76) | 0.62 (0.47-0.81) | 0.67 (0.52-0.86) | |
|  | 2017 | 0.81 (0.72-0.9) | 0.81 (0.74-0.89) | n/a | 0.67 (0.44-1.01) | 0.72 (0.55-0.94) | n/a | |
|  | 2018 | 0.81 (0.73-0.91) | 0.82 (0.75-0.91) | n/a | 0.57 (0.37-0.86) | 0.63 (0.48-0.82) | n/a | |
| **Age/Year** | | 1.04 (1.03-1.04) | 1.03 (1.03-1.03) | 1.03 (1.03-1.03) | 1.05 (1.04-1.06) | 1.03 (1.02-1.04) | 1.03 (1.02-1.03) | |
| **Sex** | |  |  |  |  |  |  | |
|  | Male | 1.00 | 1.00 | 1.00 | 1.00 | 1.00 | 1.00 | |
|  | Female | 1.03 (0.98-1.08) | 1.01 (0.97-1.06) | 1 (0.95-1.05) | 0.91 (0.75-1.11) | 0.93 (0.83-1.05) | 0.89 (0.78-1.01) | |
| **CCI** ^¶^ | |  |  |  |  |  |  | |
|  | 0 | 1.00 | 1.00 | 1.00 | 1.00 | 1.00 | 1.00 | |
|  | 1-2 | 1.08 (1-1.17) | 1.03 (0.96-1.1) | 1.02 (0.95-1.1) | 1.06 (0.82-1.37) | 0.93 (0.79-1.09) | 1 (0.84-1.18) | |
|  | ≥3 | 1.67 (1.58-1.76) | 1.53 (1.45-1.6) | 1.55 (1.46-1.63) | 1.42 (1.15-1.77) | 1.28 (1.11-1.46) | 1.31 (1.13-1.51) | |
| **Extent of Spread** | |  |  |  |  |  |  | |
|  | Non-metastatic | 1.00 | 1.00 | 1.00 | 1.00 | 1.00 | 1.00 | |
|  | Metastatic | 2.1 (1.98-2.23) | 1.87 (1.77-1.96) | 1.88 (1.78-1.99) | 2.46 (1.93-3.12) | 1.9 (1.6-2.26) | 1.77 (1.47-2.14) | |
| **Pancreatectomy** | |  |  |  |  |  |  | |
|  | No | 1.00 | 1.00 | 1.00 |  |  |  | |
|  | Yes | 0.24 (0.22-0.27) | 0.35 (0.33-0.38) | 0.4 (0.38-0.44) |  |  |  | |
| **Hospital volume** | |  |  |  |  |  |  | |
|  | Low (≤5) | |  |  | 1.00 | 1.00 | 1.00 | |
|  | Medium (6-15) | |  |  | 0.99 (0.68-1.43) | 1.05 (0.82-1.35) | 1.04 (0.8-1.34) | |
|  | High (≥16) | |  |  | 0.85 (0.59-1.22) | 1.1 (0.86-1.4) | 1.12 (0.87-1.43) | |
| **Hospital peer group^††^** | | |  |  |  |  |  | |
|  | Principal referral |  |  |  | 1.00 | 1.00 | 1.00 | |
|  | Major |  |  |  | 0.96 (0.63-1.45) | 1.13 (0.86-1.5) | 1.15 (0.87-1.53) | |
|  | Private |  |  |  | 0.78 (0.63-0.97) | 0.9 (0.79-1.03) | 0.94 (0.81-1.07) | |

Notes: ^†^ (n=3,772) excluded from analysis due to incomplete data, (n=9,774) analysed; ^‡^Individuals diagnosed in 2017-2018 were not included due to lack of five year follow up. (n=2,919) excluded from analysis due to incomplete data, (n=7,483) analysed; ^§^  (n=298) excluded from analysis due to incomplete data, (n=2,141) analysed; ^||^Individuals diagnosed in 2017-2018 were not included due to lack of five year follow up. (n=205) excluded from analysis due to incomplete data, (n=1,585) analysed; ^¶^Charlson Comorbidity Index (CCI) score. ^††^Principal referral: volume of greater than 35,000 separations and offering highly specialised services; Major: volume of greater than 10,000 separations.
